# NADPH Oxidases: Insights into Selected Functions and Mechanisms of Action in Cancer and Stem Cells

**DOI:** 10.1155/2017/9420539

**Published:** 2017-05-23

**Authors:** Magdalena Skonieczna, Tomasz Hejmo, Aleksandra Poterala-Hejmo, Artur Cieslar-Pobuda, Rafal J. Buldak

**Affiliations:** ^1^Institute of Automatic Control, Silesian University of Technology, Akademicka 16, 44-100 Gliwice, Poland; ^2^Biotechnology Centre, Silesian University of Technology, Krzywoustego 8, 44-100 Gliwice, Poland; ^3^Department of Biochemistry, School of Medicine with the Division of Dentistry, Medical University of Silesia, Jordana 19, 41-808 Zabrze, Poland; ^4^Stem Cell Group, Nordic EMBL Partnership, Centre for Molecular Medicine Norway (NCMM), University of Oslo, P.O. Box 1137, Blindern, 0318 Oslo, Norway

## Abstract

NADPH oxidases (NOX) are reactive oxygen species- (ROS-) generating enzymes regulating numerous redox-dependent signaling pathways. NOX are important regulators of cell differentiation, growth, and proliferation and of mechanisms, important for a wide range of processes from embryonic development, through tissue regeneration to the development and spread of cancer. In this review, we discuss the roles of NOX and NOX-derived ROS in the functioning of stem cells and cancer stem cells and in selected aspects of cancer cell physiology. Understanding the functions and complex activities of NOX is important for the application of stem cells in tissue engineering, regenerative medicine, and development of new therapies toward invasive forms of cancers.

## 1. Introduction

Reactive oxygen species (ROS) are highly reactive oxygen-derived molecules that include free radicals such as superoxide or hydroxyl radicals, as well as nonradicals such as ozone or hydrogen peroxide. Depending on their level, ROS can play dual roles either as important mediators and signaling molecules required for proper cell functioning or as damaging factors leading to mutations, carcinogenesis, and cell death.

To keep the correct equilibrium between the production of ROS and their elimination, free radical scavengers, both endo- and exogenous, are needed. It has been commonly believed that antioxidants which neutralize ROS and thus protect biomolecules from damage should be beneficial in protection against cancer, but recent studies clearly show that antioxidants (in the form of dietary supplements) may actually promote tumor growth and cancer metastasis. In 2011, it was demonstrated, during a trial on over 30,000 men over 50 who were administrated high doses of vitamin E, that the risk of prostate cancer increased by 17% [1]. More recently, researchers from Sweden have shown that even relatively low doses of antioxidants may enhance the growth of lung tumors and melanomas in mice [2, 3]. Similar conclusions come from work which demonstrated that treating melanoma-bearing mice with antioxidants decreased oxidative stress in circulating cancer cells but increased their ability to metastasize [4]. No matter how puzzling or confusing these evidences are, it is undoubtedly important to understand better the biology of ROS and their sources to effectively treat various diseases and disorders.

The main sources of ROS in cells, beside the respiratory chain, are NADPH oxidases (NOX). The physiological functions of NADPH oxidases are very diverse: they play a role in cellular proliferation, serotonin biosynthesis, endothelial signaling, regulation of renal functions, and the immune response against microorganisms (as a source of the so called oxidative burst), but their overexpression is associated with various neurological diseases and cancer types [5–8].

The roles of NOX have been quite well established in many noncancerous cells, but the effects of NOX-generated ROS on functioning of cancer and stem cells are much less understood. Considering the role of ROS in cancer recurrence and chemo- and radiotherapy resistance, this seems to be one of the most important research areas in the current oxidative medicine [9]. Here, we review the importance of NOX and NOX-derived ROS in the functioning of stem cells, including cancer stem cells, and in cancer cells, focusing on their roles in differentiation, self-renewal, proliferation, angiogenesis, and metastasis (Table 1).

## 2. NADPH Oxidases

**Figure 1:**
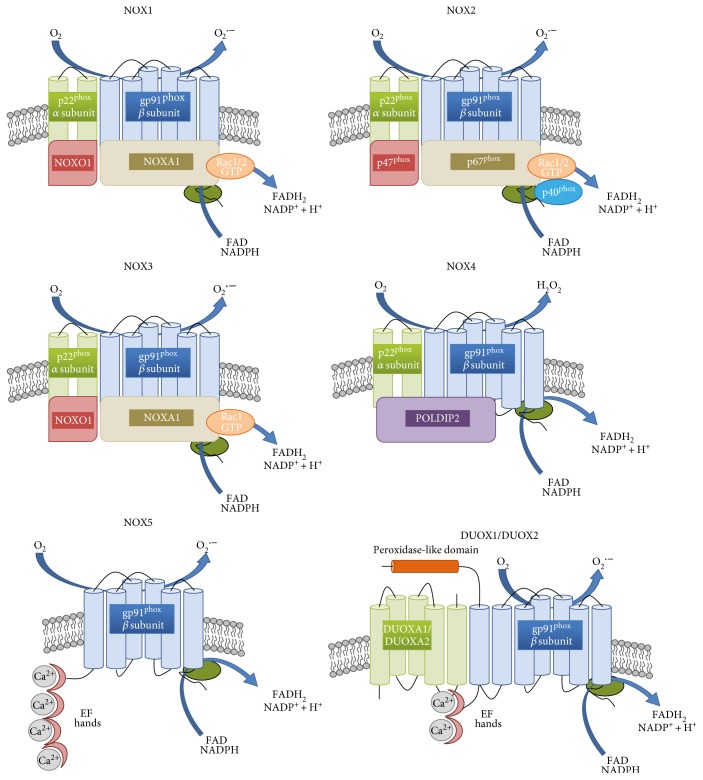
Structure of NOX isoforms. NOX1–4 are similar in their size and domain structure. Two NOX subunits, gp91phox and p22phox (also called *β* and *α* subunits, resp.), are integral membrane proteins that together comprise the large heterodimeric subunit flavocytochrome b558 (cyt b558). The cytoplasmic C-terminus contains flavin adenine dinucleotide (FAD) and NADPH-binding domains (shown in the picture as a green ellipse). NOX1 and NOX2 activation involves the phosphorylation of NOXO1 and p47phox, respectively, the translocation of the entire multidomain complex, including p40phox, p67Phox, and Rac from the cytosol to the membrane, and the transfer of electrons from the substrate to oxygen. Like NOX1 and NOX2, NOX3 is p22phox dependent, but it does not bind to Rac. NOX4 activation involves p22phox and POLDIP2. NOX5, DUOX1, and DUOX2 have calcium-binding regions (EF hands) at their N-terminus, which distinguish them from other NOX. DUOX1 and 2 have a domain with a structure similar to the active site of peroxidase but without peroxidase or superoxide dismutase activity.

The NOX family is a group of transmembrane proteins able to transport electrons from NADPH and to reduce oxygen to the ROS superoxide anion (O_2_^·−^) and hydrogen peroxide (H_2_O_2_) [10]. NOX and the mitochondrial electron transport chain are considered as the main sources of ROS in cells, although other potential sources such as cytochrome p450, xanthine oxidase (XO), or nitric oxide synthase (NOS) also contribute to the redox potential [11]. In mammals, seven proteins with NOX activity exist, NOX1 to NOX5 and Duox1 (dual oxidase 1) and Duox2; however, rats and mice lack NOX5 [12]. NADPH oxidases can be found not only within the plasma membrane (NOX1–5 and DUOX1-2) but also in the endoplasmic reticulum (NOX2, NOX4, and NOX5), mitochondrial membrane (NOX4), and nuclear membrane (NOX4 and NOX5), as well as in the specialized membrane microdomains caveoli and lipid rafts (NOX1), focal adhesions (NOX4), and invadopodia (NOX1 and NOX4) [13–19]. Every NOX family member is anchored to the membrane through six transmembrane helices binding two haem cofactors [20]. The C-terminal domain binds FAD/NADPH and allows electron transfer to the haem and further across the membrane to molecular oxygen [13, 21, 22]. DUOX1, DUOX2, and NOX5 also have calcium-binding regions at their N-terminus, which distinguish them from other NOX. Additionally, DUOX1 and 2 possess a domain with a structure similar to the active site of peroxidase (but do not show peroxidase or superoxide dismutase activity) [23, 24]. NOX1, NOX2, NOX3, and NOX5 produce O_2_^·−^ while NOX4, DUOX1, and DUOX2 generate H_2_O_2_ (see Figure 1()) [22]. To create an active NOX complex, stable complex NOX1–3 require binding to a membrane protein p22phox, cytosolic proteins p47phox, p67phox (or their homologues named NOXO1 and NOXA1, resp.), p40phox (only for NOX2), or the GTP-binding protein Rac1/2 [21, 25–28]. The main role of these subunits is to bring FAD and NADPH close together to facilitate the transport of electrons [29]. NOX4 interacts with p22phox but not with other proteins, and therefore, it is believed to be constitutively active and regulated at the level of transcript expression, or the activation may occur in a yet unknown way [30–32]. The activity of NOX4 may be enhanced by an interaction with DNA polymerase-*δ*-interacting protein 2 (POLDIP2) [33, 34]. DUOX1, DUOX2, and NOX5 activation is independent from cytosolic subunits—they contain EF-hands (helix-loop-helix motifs) that bind calcium ions for activation [12, 35, 36]. The mechanism of the activation of NOX family enzymes has been described in detail in [26, 30].

Once the active NOX complex is formed, electrons are transferred from NADPH to FAD, causing its reduction to FADH_2_ [13]. As the NOX catalytic subunit can accept only one electron, a single electron is passed to the first inner haem and then used for the reduction of molecular oxygen bound by the second haem [10, 37]. Superoxide anion generated in this reaction often undergoes disproportionation reactions in which one molecule of O_2_^·^ donates an electron to another, forming H_2_O_2_ and O_2_ in a reaction termed dismutation (catalyzed by superoxide dismutase (SOD) or occurring spontaneously under low pH conditions) [38]. As described above, H_2_O_2_, rather than superoxide anion, has been identified as a product of NOX4, DUOX1, and DUOX2 but it is predicted that for thermodynamic reasons, this cannot be formed through haem-catalyzed two-electron reduction [13, 39]. More likely, some regions in NOX4, DUOX1, and DUOX2 serve as enhancers of spontaneous dismutation or as a proton donor, but this hypothesis has not been confirmed [13, 40].

**Figure 2:**
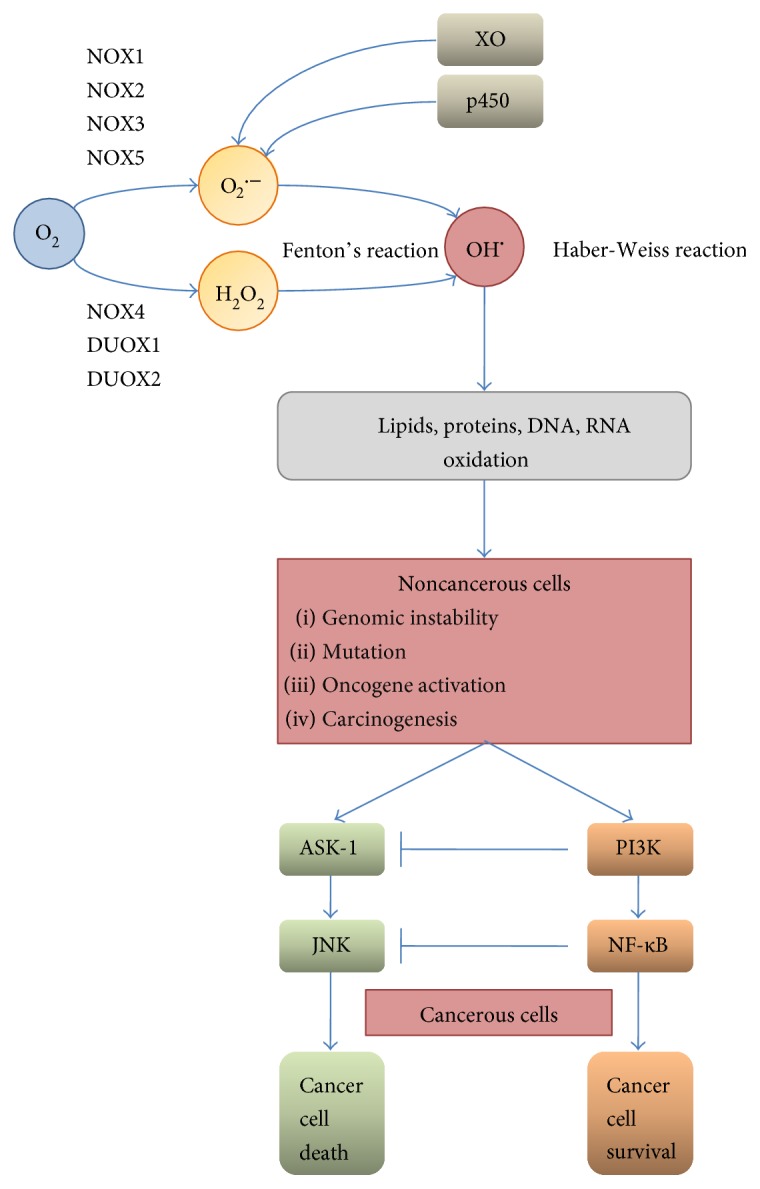
Endogenous sources and consequences of ROS overproduction. Overproduction of superoxide anion and H_2_O_2_ by NOX (NOX and Duox), cytochrome c oxidase, or xanthine oxidase (XO) and the subsequent increased level of hydroxyl radical (generated through the Fenton or Haber-Weiss reaction) are leading to lipids, proteins, and nucleic acid oxidation and, in consequence, to genomic instability, mutations, and carcinogenesis. Upon this conditions, cell survival or death is dependent on the activation of either ASK-1 or PI3K: high level of ROS tends to activate the ASK-1/JNK pathway leading to cell death, while lower or transient ROS production may result in the activation of PI3K kinase (accompanied by ASK-1/JNK inhibition) and NF-*κ*B-mediated survival.

ROS, including NOX-derived superoxide (O_2_^·^) and H_2_O_2_, inhibit the activities of various biological molecules. At low levels, they serve as the second messengers for signal transduction, but higher concentrations cause oxidative damage to DNA, proteins, and lipids by direct oxidation or via the transition metal-driven Haber-Weiss reaction to the extremely reactive hydroxyl radical (OH^·^) (Figure 2) [41–44].

H_2_O_2_ induces apoptosis in many cancer cells in vitro via the activation of the caspase cascade. Many antitumor drugs, such as vinblastine, doxorubicin, or camptothecin, also exhibit antitumor activity via H_2_O_2_-dependent activation of apoptotic cell death, which suggests the potential use of H_2_O_2_ as an antitumor agent [45–48]. However, a biphasic effect of H_2_O_2_ and superdoxide has been demonstrated on cellular proliferation in which low levels (submicromolar concentrations) induce growth, but higher concentrations (>10–30 micromolar) induce apoptosis or necrosis. This phenomenon has been demonstrated for primary, immortalized and transformed cell types [38, 44].

This review highlights the relations between NOX proteins and several cellular processes which are of importance in medicine and hallmarks of cancer such as increased proliferation rate, avoidance of apoptosis, tumor invasiveness, tumor angiogenesis, differentiation, and self-renewal of stem cells, showing that several NOX may be considered as potential therapeutic targets. The expression of NOX, especially in cancer cells and solid tumors, has been a topic of several publications in the past few years (for details, see [11, 49]). Therefore, we concentrate here on selected processes in which NOX have been identified as important players, particularly proliferation, invasiveness, and metastasis, paying particular attention to potential mechanisms of action and regulation.

## 3. Roles of NOX in Cancer Cells

Increased expression of NOX1, NOX2, NOX4, and NOX5 or their regulatory components compared with normal controls has been described in many types of cultured cancer cell lines and in human tumors at early and late stages of tumorigenesis, indicating their importance in cancer development and progression [49–51].

Exogenous expression of NOX1 in NIH3T3 fibroblasts caused increased cell growth and the ability to form tumors in athymic mice [52]. Tenfold overexpression of NOX1 caused increased growth and transformation with only a < 2-fold increase in extracellular O_2_^·-^ generation, showing that high levels of ROS are not required for these effects. Coexpression of catalase (CAT) reversed the transformed phenotype, indicating that H_2_O_2_ was the growth-promoting species [38, 52].

Studies of tumor and adjacent tissues from 123 patients with gastric cancer (*adenocarcinoma*) showed that in 47.2% of cases, the NOX2 level was detectable and was increased in the tumor compared to adjacent tissue. Patients in the NOX2-positive group presented a poor prognosis (5-year survival rates) [53].

The roles of NOX3, DUOX1, and DUOX2 in cancer and stem cells have not been very well established as yet. According to the current state of knowledge, NOX3 expression is generally limited to the cochlea and inner ear epithelial cells, where it plays a role in the perception of gravity and maintaining balance [54, 55]. DUOX promoters have been shown to be highly methylated in lung cancer [54, 56]. Recently, it has been also demonstrated that the loss of DUOX1 expression in lung cancer cell lines is strongly associated with the loss of the epithelial marker E-cadherin and that the silencing of DUOX1 promotes features of an epithelial-to-mesenchymal transition (EMT), an important feature of metastatic cancer [57]. Wu et al. [54] showed that DUOX2 is regulated by IFN-*γ*-mediated Stat1 binding to the Duox2 promoter in pancreatic tumor lines. At the same time, the authors demonstrated the upregulation of DUOX2 expression in vivo in pancreatic cancer xenografts and in patients with chronic pancreatitis. In another study by Wu et al. [58], IFN-*γ*-mediated DUOX2 overexpression resulted in H2O2-induced, ERK-associated upregulation of HIF-1*α* and VEGF-A in pancreatic cancer cells. Recently, DUOX enzymes were also found to constitutively maintain ROS levels in prostate cancer cells, and these ROS promote AKT signaling leading to increased resistance to apoptosis [59].

### 3.1. NOX in Tumor Development

Oxidative stress can result in genomic instability caused by direct modification and damage to nucleic acids and alteration of redox-sensitive proteins and signal transduction leading to tumor formation. The exact function of NOX-derived ROS in cellular transformation remains an open question.

ROS produced by NOX4 caused mitochondrial dysfunction and mitochondrial DNA damage [15, 60, 61]. As NOX4 can be found also in the nucleus, it could be responsible for direct oxidation of nuclear proteins and DNA as well [62]. Whether NOX play a role in ROS-induced genomic instability resulting in tumorigenesis is uncertain, as this would be an outcome of complex interactions between reactive species, antioxidants, and DNA repair pathways.

Besides, genomic instability NOX1 has been connected with the regulation of p53 activity. The corepressor HIPK2 which control the tumor suppressor p53 also upregulates NOX1, which in turn prevents deacetylation and inactivation of p53 by the stress-controlling protein sirtuin1 (SIRT1), therefore promoting tumorigenesis [63, 64].

Increased expression of NOX1 also accompanies activating mutations in K-RAS, a proto-oncogene with a key role in growth autonomy of tumor cells [65]. Overexpression of K-RAS enhances the transcription of NOX1 through RAF/MEK/ERK-dependent phosphorylation of the transcription factor GATA6 [66]. In RAS-transformed cells, NOX1 stimulates cell proliferation and anchorage-independent growth through the RAS/MEK and canonical WNT-*β*-catenin pathways [67, 68]. NOX regulate several other phosphatases linked to cell survival, for example, the low molecular weight protein tyrosine phosphatases (LMW-PTP) and protein phosphatase 1 (PP1). In chronic myeloid leukemia, increased NOX4-mediated ROS production induced by Bcr-Abl (kinase generated from the Philadelphia chromosome) enhances survival signal transduction through the inhibition of PP1 which negatively regulates the PI3k/Akt pathway [69].

#### 3.1.1. NOX in Proliferation, Invasiveness, and Metastasis

All cancer cells share some features known as the hallmarks of cancer which include insensitivity to growth-inhibitory signals, limitless replicative potential, self-sufficiency in growth signals, avoidance of apoptosis, sustained angiogenesis, tissue invasion, and metastasis [70, 71].

Recent studies on colon cancer show that NOX1 activity and ROS generation are modulated through a cascade of interactions between growth receptor-bound protein 2 (an adaptor involved in signal transduction), Cbl E3 ligase (an ubiquitin-protein ligase), NOXA1 (an activator of the NOX complex), epidermal growth factor, and NOXO1 (an organizer of the NOX complex). NOX1 can modulate the canonical Wnt-*β*-catenin signaling pathway which is crucial for the proliferation and fate of both malignant and normal cells, and on the other hand, Wnt can induce NOX1-derived ROS production [67]. Thus, the connections between NOX activity and cancer cell growth, proliferation, and tumor formation involve complex signaling pathways and interactions.

Cancer cells can be transferred to and proliferate in different regions of the same organ or in very distant sites. This spreading, termed metastasis, is a multistep process involving the invasion of tumor cells into the extracellular matrix, the migration through the endothelium into vessels (intravasation) and then out of them (extravasation), and finally, the colonization and proliferation leading to growth of the secondary tumors [72, 73]. The steps of extracellular matrix degradation and extravasation are mediated by invadopodia, actin-rich structures in the plasma membrane which contain integrins, matrix metalloproteinases, NOX, and other transmembrane proteins. The formation of invadopodia is dependent on NOX-mediated production of ROS [17, 74, 75]. Two proteins found only invadopodia, named Tks4 and Tks5, show homology to p47phox (as well as some structural similarities to p40phox and NOXO1 and associate with and activate NOX1 and NOX3, even when no other organizers were present [76, 77]. This suggest that ROS-dependent invadopodia formation may be dependent on a NOX-Tks protein complex, formed exclusively at invadopodia membranes [17, 78].

Higher metastatic potential is correlated with elevated matrix metalloproteinase-7 (MMP-7) expression in human colon cancer cells; less invasive colon cancer cells, such as Caco2 and HT29 cell line, present low MMP-7 expression level and high NOX1 and AMPK phosphorylation levels. AMPK is an energy-sensing kinase, activated by changes of the AMP/ATP ratio caused by insufficient amount of nutrients and required for rapid cell proliferation and hypoxic conditions. Pharmacological activation of AMPK by adding 5-aminoimidazole-4-carboxamide riboside (AICAR) and D942 leads to reduced NOX4 expression and NOX4-dependent ROS generation. NOX may be linked to inflammation-induced metastasis in renal cell carcinoma (RCC), where cell invasion is based on NOX4-mediated hypoxic-induced production of interleukin-6 and interleukin-8 (IL-6 and IL-8) [79]. IL-6 and IL-8 induce metastasis in RCC, but their NOX-dependent production can be reduced through the activation of AMPK (previously shown to decrease tumor growth in a xenograft model and in vitro) [79, 80].

The induction of an invasive phenotype by TPA (12-O-tetradecanoylphorbol-13-acetate) results in increased NOX2 and MMP-7 expression which leads to higher ROS production and decreased AMPK phosphorylation [81]. In colon cancer cells, this molecular switch from NOX1 to NOX2, together with NOX2-derived ROS, increases MMP-7 expression by the deactivation of AMPK, and the TPA-induced phenotype can be reverted by NOX2 but not NOX1-targeting siRNA, suggesting that NOX2 activity induces an invasive phenotype [81]. In contrast, in melanoma cells, the role of a switch-deciding transformation from a noninvasive to an invasive phenotype is played by the Akt protein, a kinase that plays a key role in proliferation, cell migration, and apoptosis, which induces NOX4-derived ROS [82].

The inhibition of NOX by siRNA or a pharmacologic inhibitor leads to significantly reduced lung cancer formation in vivo and lung cancer cell invasion in vitro, as shown by a meta-analysis [83]. NOX1-derived ROS are crucial for the regulation of metastasis through the toll-like receptor 4 (TLR4) in non-small lung cancer cells (NSCLC) [84], and it is possible that TLR4 signaling enhances the expression of NOX1 which subsequently regulates MMP-9 and increases metastasis in these cells [84, 85]. O'Leary described the role of TLR-4-dependent NOX1 activity in accelerating adherence of lipopolysaccharide- (LPS-) stimulated colon cancer cells (SW480, SW620, and CT-26 cell lines) and proposed a mechanism in which TLR-4-mediated activation of NF-*κ*B leads to increased activation of NOX and in consequence to a higher level of ROS and phosphorylation of Akt [86]. The PI3K/Akt signaling pathway mediates TG-interacting factor- (TGIF-) induced NOX2 activation and ROS production, which stimulate PI3K/Akt to promote the invasiveness of urothelial *carcinoma* [87]. TGIF acts as a transcriptional repressor/corepressor regulated by TGF-*β* and associated with the protein SMAD [88]. TGF-*β*/SMAD3-induced NOX4 activity affects cell migration and expression of fibronectin, a marker of TGF-*β*-induced epithelial-to-mesenchymal transition (EMT), in normal and metastatic breast epithelial cells [89, 90].

There is growing body of evidence that microRNAs are involved in NOX-dependent regulation of tumor growth, invasiveness, and metastasis [91–93]. miR-21 has been reported to promote growth, metastasis, and chemo- and radioresistance in non-small lung cancer cells by targeting PTEN, the product of a tumor suppressor that is mutated in many cancers [93]. The inhibition of NOX activity in human lung cancer cells decreases their invasive potential in vitro, lowers the level of miR-21 and MMP-9, and also increases expression of PTEN. The expression of miR-21 and of the NOX subunit p47phox was significantly higher in poorly differentiated tumor cells. An increased level of miR-21 also compensates for the effect of NOX inhibitors on metastasis [92]. Consistent with the results for prostate cancer cells in which NOX-derived ROS are known to regulate invasiveness and metastasis in vivo, the depletion of p47phox subunit using siRNA reduced tumor metastasis in a xenograft model of prostate cancer [94]. The regulation of miR-21 by NOX-derived ROS probably occurs by the activation of Akt, but understanding of this axis requires further studies [94, 95]. miRNAs can also affect the NOX4-MCP-1 axis critical for *hemangioendothelioma*; silencing of the enzyme required for miRNA maturation (Dicer) prevented formation of tumors in vivo, accompanied by the upregulation of miR-21a-3p activity targeting the 3′UTR of the NOX4 transcript [96]. A lower level of NOX4-derived ROS resulted in decreased production of the oxidant-inducible monocyte chemoattractant protein-1 (MCP-1) which is critical for endothelial cell tumor formation [96, 97].

The role of NOX5 is less understood than those of NOX1, 2, and 4, but it is also reported to play a significant function in cancer. Its silencing results in a lower proliferation rate of prostate cancer PC-3 cells and increased apoptosis caused by enhanced activity of caspases 3 and 7 [51], and therefore, NOX5-derived ROS are suggested to be important for the regulation of proliferation and survival of prostate cancer cells [51]. Lower expression of NOX5 resulted in a decreased level of phosphorylation of c-Jun N-terminal kinase 1/3 (JNK1/3) and a reduced level of PKC-*ζ* protein, which is known to promote an aggressive phenotype of human prostate cancer cells [51, 98].

#### 3.1.2. NOX and Angiogenesis

Cell function and survival and, in the case of cancer cells, also the ability to spread to adjacent and distant tissues are dependent on the oxygen and nutrients provided by the vasculature [99]. Angiogenesis is a critical step for the development of tumors with a diameter higher than ~2 mm as it allows the delivery of nutrients into the solid tumor [13, 99]. New blood vessel formation from existing vasculature is regulated by growth factors such as vascular endothelial growth factor (VEGF) which activates matrix metaloproteinases (MMPs) and can be regulated by inhibitors (i.e., angiostatin) [100]. Another key element of angiogenesis in tumors is the transcription factor hypoxia-inducible factor 1 (HIF-1) which under hypoxic condition characteristic for the center of tumor, increases the transcription of VEGF, and furthermore, both HIF-1*α* and VEGF expression can be stimulated by ROS [100, 101].

**Figure 3:**
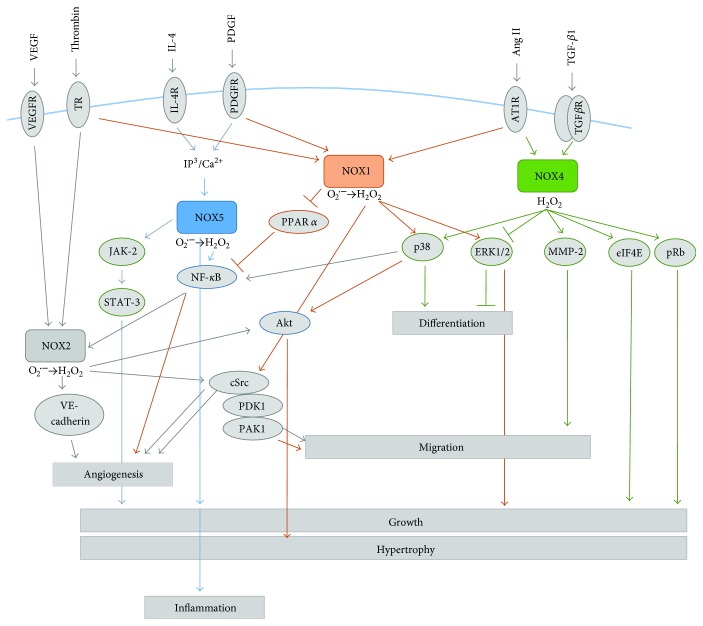
Examples of NOX signal transduction. NOX1-derived ROS can affect the following: differentiation through the p38 and ERK1/2 pathway, hypertrophy via p38-mediated activation of Akt, cell migration by the activation of cSrc protein, and cellular growth by the activation of ERK1/2; and subsequent activation of transcription factor Ets-1 and upregulation of cyclin *D. nox*1 also downregulates the expression and activity of the antiangiogenic receptor PPAR*α*, known to inhibit the transcription factor NF-*κ*B and, therefore, affects angiogenesis. NOX1 can be activated by thrombin, PDGF, or Ang II; activated by TNF*α*, thrombin or NF-*κ*B NOX2-generated ROS promote migration and angiogenesis through the Akt and cSrc pathways. NOX2-mediated regulation of angiogenesis also occurs via VE-cadherin; via the activation of p38 or the inhibition of ERK1/2 NOX4-derived ROS promotion; via the activation of p38 or the inhibition of ERK1/2 differentiation, which regulates growth, by eiF4E and pRB pathways, and migration (through the activation of MMP2). NOX4 expression and activity can be increased by TGF-*β*1 or Ang II; NOX5 promotes growth and inflammation, respectively, through JAK-2/STAT3 and NF-*κ*B signaling pathways. ROS production by NOX5 can be activated by IL4 or PDGF; there will be always some omitted proteins, no matter how detailed is the scheme.

In ovarian cancer cells, NOX4-derived ROS together with mitochondrial-derived ROS are necessary for tumor-induced angiogenesis and regulation of VEGF level through HIF-1*α* expression [13, 102]. NOX1- and NOX4-derived ROS promote HIF-dependent vascularization in prostate cancer and malignant melanomas; however, several reports indicate that ROS-mediated angiogenesis can also occur through an HIF-independent mechanism [13, 82, 103]. Garrido-Urbani et al. reported increased expression and activity of NOX1 during angiogenesis and impaired angiogenesis in NOX1-deficient mice, indicating its role in endothelial cell migration and tumor progression [104]. NOX1 downregulates expression and activity of the antiangiogenic receptor PPAR*α* (peroxisome proliferator-activated receptor *α*) which is known to inhibit the transcription factor NF-*κ*B (see Figure 3) and VEGF [104, 105]. Another mechanism has been reported for serotonin-induced angiogenesis: serotonin (5-HT, 5-hydroxytryptamine) activates NOX and induces ROS production, which is probably mediated through the activation of the 5-HT1 receptor-linked Src/PI3K pathway [106]. However, it is not clear if HIF-1 plays a role in this mechanism because the PI3K pathway can increase VEGF production by tumor cells in both an HIF-1-dependent and HIF-1-independent manner [106, 107].

## 4. NOX in Stem Cells

Stem cells play important roles in many stages of development, from progenitors of all cells, pluripotent stem cells, in early embryonic stages, to tissue-restricted cells—giving rise to cells with highly specialized functions [108]. Numerous studies have demonstrated the potential of stem cells in therapies [109]. Redox states have been reported to play important role in both maintaining stemness and mediating differentiation of several precursor cell types [110].

### 4.1. NOX and Differentiation

NOX2 regulates the differentiation of mouse induced-pluripotent stem cells (miPCs) into arterial endothelial cells (miPSC-ECs) via the Notch signaling pathway [111]. The expression of arterial endothelial markers such as EphrinB2, neuropilin 1 (Nrp1), and activin receptor-like kinase 1 (ALK1), as well as the expression of Notch-pathway components, was significantly decreased (at the mRNA and protein levels) in NOX2^−/−^ miPSCs. However, the transfection with an adenovirus vector coding for NOX2 resulted in a significant increase of arterial endothelial markers and Notch1 expression, and the same effect was obtained by the upregulation of Notch activity. In both cases, the effect of this increase can be reversed either by DPI-induced inhibition of ROS generation or by silencing of Notch1 expression [111]. NOX2 deficiency has been shown to significantly lower the potency of miPSC-ECs for vascular repair in mouse ischemic limbs, tube formation, cell proliferation, cell migration, and uptake of Ac-LDL (acetylated low-density lipoprotein) and to increase sensitivity to oxidative stress [111].

NOX4 has been reported to regulate *myogenesis*, the process in which muscle stem cells first proliferate and then differentiate. In myogenic C2C12, cell changes in NOX4 expression level correlate with the changes in the level of the differentiation markers myogenin, MyoD1, Pax7, and Myf5 which can be further linked to the changes in MAPK signaling pathways. Both overexpression and depletion of NOX4 caused reduction of ERK1/2 phosphorylation during the differentiation [112]. The MAPK family consists of extracellular regulated kinases (ERK1/2), Jun N-terminal kinase (JNK), p38 kinase, ERK3/4, and the mitogen-activated protein kinase 1 (BMK1/ERK5) pathways. The JNK and p38 kinase pathways are sometimes grouped together and referred to as the stress-activated protein kinases (SAPKs) [113]. NOX are also implicated in the differentiation of cardiac cells into cardiac muscle, endothelial, and smooth muscle cells. Cardiac precursor cells (CPCs) marked by type III receptor tyrosine kinase c-kit (c-kit^+^) with silenced *Nox2* and *Nox4* genes showed increased expression of the CPC stemness markers c-kit and Flk1 (receptor for vascular endothelial growth factor), while cells with overexpression of NOX2 and NOX4 presented decreased c-kit level [114].

These changes were accompanied by changes in the level of Gata6, Gata4, and cytokine-transforming growth factor *β*1 (TGF-*β*1) required for cardiac lineage specification, as well as an altered level of the differentiation markers *α*-smooth muscle actin (*α*-SMA) and cardiac troponin T (cTnT). The upregulation of NOX2 and NOX4 during the differentiation of early postnatal c-kit^+^ cells suggests that they are responsible for maintaining “the balance between precursors and differentiation status” [114]. NOX4 has also been described as a mediator of the differentiation of mouse embryonic stem cells into smooth muscle cells (SMC) with a positive correlation between the expression of NOX4 and SMC-specific genes (*SMαA*, *SM22α*, *h1-colponin*, and *SM-myh11*) and transcription factors essential for the differentiation (serum response factor and myocardin), expression, and activation of NOX4 (which can occur through TGF-*β*1) driving the differentiation (and maintenance of phenotype) of functional SMC from EC through H_2_O_2_ generation [115].

NOX4 activity is essential for BMP-induced neuronal differentiation of neural crest stem cells (NCSCs) [116], differentiation of osteoblasts from murine 2T3 preosteoblast cells [117], and profibrotic cell differentiation from adult renal progenitor cells [118]. The silencing of NOX4 in primary NCSCs leads to cell death; however, in NOX4^−/−^ knockout mice, the development of the neural crest-derived peripheral nervous system occurred normally (although embryos showed retarded growth). As NOX4 is the only NOX expressed in NCSCs at a detectable level, it is suggested that other NOX proteins from surrounding cells and tissues may provide ROS for NCSCs during embryogenesis [116]. It seems also that NOX levels and functions are dynamically regulated during mouse embryonic stem cell differentiation, as p67phox subunit expression is significantly increased in 2-3-day-old embryoid bodies compared to those 11-12-day old [119, 120].

### 4.2. NOX and Stem Cells Self-Renewal

There are two key characteristics of stemness: one is potency, understood as an ability to differentiate, and the other is self-renewal, an ability to maintain a pool of undifferentiated stem cells through symmetric and asymmetric cell divisions [121]. The stimulation of NOX4-derived superoxide production by angiotensin II (Ang II) in neural stem cells significantly increases their proliferation [122], leading to the suggestion that NOX4 level regulates stem cell self-renewal and, therefore, may be an important player in neurodegenerative processes such as Alzhaimer's disease, Parkinson's disease, or multiple sclerosis [122]. A function of NOX in self-renewal has also been reported by Morimoto et al. who described a connection between the self-renewal potential of mouse spermatogonial stem cells (SSCs) and NOX3 and presented the hypothesis that renewal of SSCs is in fact regulated by sequential activation of different NOX genes and may or may not occur through the PIK3-AKT and MAP2K1 pathways [123]. Similar results were obtained for adipose-derived stem cells (ASCs) in which the silencing of NOX4 leads to reduced proliferation and cell migration, as well as decreased expression of Oct4 and Rex1 and a lower level of phosphorylation of PDGFR-*β*, AKT, and ERK1/2 [124].

In contrast, another study suggests that the suppression of NOX using apocynin can reverse the aging process in mesenchymal stem cells and increases the expression of the transcription factors Nanog and Oct-4—which are important in the self-renewal of stem cells [125]. Increased expression of NOX2 and NOX4 has been reported to accelerate senescence of Ang II-stimulated endothelial progenitor cells [126].

ROS generated by NOX (and other sources) can act as an enhancer of stem cell signaling but also as damaging molecules, and therefore, more studies are still required as the threshold is not clearly defined [9]. Additionally, it should be noted that the idea of NOX-mediating differentiation, aging, and senescence of stem cells interferes with data on their role in undifferentiated and not senescent cancer cells. Unfortunately, there is not enough data to draw precise conclusions.

### 4.3. NOX and Cancer Stem Cells

Several reports indicate that the growth of some solid tumors and their high resistance to chemotherapy are dependent on small population of cells called cancer stem cells (CSCs) [127].

CSCs are subgroup of cancer cells having the ability of self-renewal and capability to initiate tumor formation and metastasis [59]. So far, these tumor-initiating cells have been found in many types of cancer, breast, brain, skin, head and neck, thyroid, cervix, retina, and lung and from leukemia and lymphoma [128–131]. The origin of CSCs remains still unclear, and it is debated if they are formed in a process similar to reprogramming (dedifferentiation) from cells that have acquired a more stem cell-like phenotype or if they are stem cells which have accumulated the sufficient number of mutations required for carcinogenic transformation. It is also possible that they are a result of a combination of both these processes. The issue of the origin of these cells raises numerous discussions, and to highlight this uncertainty, they are often referred as cancer stem-like cells or tumor-initiating cells.

CSCs reside preferentially in special microenvironmental niches within tumor tissue, where cells have a limited access to oxygen. The features of these hypoxic niches are of crucial importance for CSC self-renewal, metastatic potential, and therapy resistance. CSCs express increased levels of antiapoptotic proteins in comparison to mature cell types from the same tissue, which could explain their resistance to cytotoxic drugs [132, 133].

The presence of CSCs might also explain why cancer may reoccur after treatment; most chemotherapeutic drugs act only on mature cancer cells, and CSCs have elevated levels of an ATP-binding cassette which promotes drug resistance [134]. In glioma cells, CSCs preferentially activate DNA-damage checkpoints, so the cells can repair the damage faster and escape radiation-mediated cell death [135]. In breast cancers, CSCs express a low level of ROS due to higher expression of free radical scavengers, which ultimately causes resistant to radiation therapy [136].

Recently, NOX-mediated production of ROS has been recognized as an important factor involved in cancer stem cell regulation and chemotherapy resistance [137]. Enrichment of a population of breast cancer stem-like cell population, induced by exposure to low concentrations of combined carcinogens, correlates with the activation of the RAS-Erk1/2-NOX1 pathway which plays an important role in maintaining increased cell proliferation [138]. The opposite result was observed for stem-like holoclones derived from the PC3 human prostate cancer cell line, which showed reduced expression of NOX2, NOX4, and NOX5, and their upregulation significantly lowered cell survival in vitro [139].

Gemcitabine, a chemotherapeutic drug used in advanced pancreatic cancer, is characterized by low efficiency and causes rapid development of chemoresistance [137, 140, 141]. New data show that the pancreatic cancer stem cell phenotype (characterized by CD44^+^, CD24^+^, and CD133^+^ markers) can be actually induced by gemcitabine itself [142]. Gemcitabine activates the NF-*κ*B/STAT3 signaling cascade through NOX-mediated production of ROS [137], and pancreatic cell lines incubated with the NOX inhibitor apocynin show not only a decrease in ROS and p-STAT3 levels but also an abolished expression of Nanog, Sox2, and Bmi1, genes associated with self-renewal and maintaining pluripotency [137].

NOX2 has been suggested as a potential target in the development of a therapy against chronic myeloid leukemia (CML) and glioblastoma, as the resistance of CML stem cells and patient-derived glioblastoma stem cells to tyrosine kinase inhibitors seem to be mediated through the NOX2/Egr1/Fyn axis [143]. Chemotherapy may induce the overproduction of ROS which leads to NF-*κ*B-mediated release of inflammatory cytokines, including IL-6 and IL-8, and drive cancer progression through inflammation. Additionally, interleukin-6 is known to induce resistance of myeloma cells to chemo- and radiotherapy by NF-*κ*B-dependent increase of manganese superoxide dismutase expression (MnSOD) [144]. Unfortunately, there is not enough data to confirm whether such re-establishing of redox homeostasis exist in cancer stem cells [144].

The role of NOX-generated ROS in the functioning of cancer stem cells is not well understood yet, but considering their role in cancer recurrence and chemo- and radiotherapy resistance, this seems to be one of the most important research areas in current oxidative medicine [9].

## 5. Summary

NOX are the only enzymes for which the production of ROS is main physiological function. They play roles in many processes required for functioning of cells and organisms, including wound healing, host defense, cell differentiation during embryogenesis, proliferation, and regulation of gene expression. However, their pathologically altered expression and activity are connected with several neurodegenerative and inflammatory diseases, as well as the development and progression of cancer [145].

NOX can modulate proliferation and differentiation of stem cells which make them a potential tool and target in stem cell therapies, tissue engineering, and regenerative medicine. Studies aimed at growing functional cardiac tissue from stem cells (neonatal rat cardiomyocytes) have already shown NOX-mediated redox signaling to be crucial for neovascularization in vivo which is necessary for the generation of functional tissues [119, 146].

Altered expression of NOX has been observed in many types of cancers [147]. As recent research has brought some light into the mechanisms of NOX-derived ROS action and effects on proliferation, invasiveness, metastasis, and angiogenesis of cancer cells, NOX have been proposed as targets in therapy of lung, colon, thyroid, and prostate cancer [51, 147, 148]. Especially, the ability to prevent metastasis through the modulation of cancer stem cell growth and proliferation would bring enormous benefits for patients. However, this approach is currently limited by the lack of highly specific and validated inhibitors for different NOX enzymes which would not affect other sources of ROS. The development of specific inhibitors seems to be even more important given the poor results of therapies based on antioxidants aimed at scavenging ROS [149]. As NOX are not the only origin of ROS in cells, it is important to understand the mutual interactions between these enzymes and other ROS sources, especially the respiratory chain, to effectively regulate redox potential for therapeutic purposes.

NOX have been intensively studied over the past decade, and the results obtained have significantly increased our knowledge about their activation and the signaling pathways which they influence; however, much more research is still required, especially in vivo using animal models.

## Figures and Tables

**Table 1:** Functions and mechanisms of action of NADPH oxidases in stem cells and cancer stem cells.

Process	Expression/activity of NOX	Effects/mechanism	Reference
Differentiation	↑ NOX2	Differentiation of stem cells/ROS-dependent Notch signaling pathway	[111]
	↓ NOX4	Myogenesis, C2C12 differentiation/↓ ERK1/2 phosphorylation, MAP kinases	[112]
	↑ NOX2 ↑ NOX4	Cardiac precursor cells (CPCs) → ↑ c-kit(+) cells/unknown mechanism	[114]
	↑ NOX4	Differentiation of endothelial cells into smooth muscle cells/TGF*β*-1-dependent NOX4/H_2_O_2_ upregulation	[115]
	↑ NOX4	NOX4/H_2_O_2_ dependent	[116–118]
		(i) Neural crest stem cells (NCSCs) differentiation to neural cells	
		(ii) 2T3 preosteoblast differentiation	
		(iii) Renal progenitor cells differentiation to profibrotic cells	
	↓ NOX4	Neural crest stem cells (NCSCs) death or retarded growth of PNS	[116]

Stem cell self-renewal	↑ NOX4	Proliferation of neural stem cells/superoxide dependent	[122]
	↑ NOX3	↑ proliferation of mouse spermatogonial stem cells/unknown mechanism	[123]
	↓ NOX4	↓ proliferation and migration of adipose-derived stem cells (ADSCs)/↓ ERK1/2, Akt, ↓ PDGF*β*1	[124]
	↓ NOX	Proliferation of mesenchymal stem cells/↑ Nanog/Oct4 (TFs)	[125]
	NOX2 NOX4	↑ senescence of Ang. II-stimulated endothelial cells/unknown mechanism	[126]

Cancer stem cell growth and survival	NOX2	↑ proliferation of pancreatic cancer cells (SW1990 and BxPC-3)/NF-*κ*B/STAT3 activation	[137]
	↑ NOX1	↑ enrichment of breast cancer stem-like cell population/RAS/Erk1/2/NOX1 activation	[138]
	↓ NOX2, NOX4, NOX5	↑ survival of prostate stem-like cells in vitro	[139]

Cancer stem cell drug resistance	↑ NOX2	↑ resistance of patient-derived glioblastoma stem cells and chronic myeloid leukemia stem cells to tyrosine kinase inhibitors/NOX2/Egr1/Fyn upregulation	[143]

## Abbreviations

AMPK: 5'AMP-activated protein kinase
BMP-2: Bone morphogenetic protein-2
CPCs: Cardiac precursor cells
CSCs: Cancer stem cells
DPI: Diphenyleiodonium
Egr-1: Early growth response 1
FAD: Flavin adenine dinucleotide
NADPH: Nicotinamide adenine dinucleotide phosphate
NOXA1: NADPH oxidase activator 1
NOXO1: NADPH oxidase organizer 1
PDGFR-*β*: Platelet-derived growth factor receptor-*β*
ROS: Reactive oxygen species
Vav2: Guanine nucleotide exchange factor 2.
